# PReS-FINAL-2027: Cerebral demyelination and optic neuritis during treatment with eternacept and methotrexate

**DOI:** 10.1186/1546-0096-11-S2-P40

**Published:** 2013-12-05

**Authors:** AE Christensen, L Bistrup, P Toftedal

**Affiliations:** 1 Hans Christian Andersen Childrens Hospital, Odense University Hospital, Odense, Denmark

## Introduction

Demyelinating disorders have been reported in association with anti-tumor necrosis factor alpha (anti-TNF-alpha) treatment in adults. Few reports exist among children.

## Objectives

We report a case of demyelination and optic neuritis in a child treated with eternacept to increase the awareness of the possible risk of demyelination during anti-TNF-alpha treatment in children.

## Methods

Case report.

## Results

A 9 year old girl treated with methotrexate for 6 months and eternacept for 3 months because of polyarticular JIA developed loss of vision in both eyes. The weeks prior she had been extremely tired with periods of cough and fever. Bacterial- and Epstein-Barr-virus infection was absent. Her well-being improved spontaneously. Mild vitritis was diagnosed bilaterally and impaired vision was diagnosed. MR of the brain showed periventricular white matter lesions and lesions in hypothalamus. In addition signs of optic neuritis were seen. Visual evoked potential (VEP) showed delayed latency. Anti-aquaporin-4 antibodies were not present. Cerebrospinal fluid (CSF) examination was without pleocytosis and no infectious agent could be demonstrated. Oligoclonal bands in the CSF were present indicating production of gamma globulin in the central nervous system.

The girl received high-dose intravenous steroid therapy followed by oral prednisone. The treatment with etanercept was stopped. Two months later her vision was almost normal and still improving. No further neurological symptoms have developed during prednisolone tapering.

## Conclusion

Development of cerebral demyelination might be the first attack of multiple sclerosis and may be triggered by anti-TNF-alpha treatment. The changes could also be due to an inflammatory disorder caused by infection or be of auto immune origin. The role of the TNF-alpha blockade is uncertain.

## Disclosure of interest

None declared.

